# Supplementary material for: Improving private tuberculosis patient notifications in Akot Tuberculosis Unit of Vidarbha region of India: A Plan-Do-Study-Act (PDSA) based approach for enhanced public health reporting
Source: PLOS Glob Public Health. 2026 May 22;6(5):e0006480. doi: 10.1371/journal.pgph.0006480 (PMC13196973; doi:10.1371/journal.pgph.0006480)
Supplement: Attachment — Submitted filename: Rebuttal_Letter_PLOS_UmeshKawalkar.docx [file pgph.0006480.s002.docx]

Response to Reviewers

Manuscript ID: PGPH-D-25-01093
Title: Improving Private Tuberculosis (TB) Notifications in Akot Tuberculosis Unit of Vidarbha Region of India: A Plan-Do-Study-Act (PDSA) Based Approach for Enhanced Public Health Reporting
Journal: PLOS Global Public Health

Corresponding Author: Dr Umesh G. Kawalkar
Email: [umeshkawalkar01@gmail.com](mailto:umeshkawalkar01@gmail.com)

We thank the Editor and all reviewers for their detailed critique and constructive feedback. All comments were reviewed meticulously. Substantial revisions were made to ensure internal consistency, data accuracy, improved readability, and alignment with PLOS Global Public Health standards. Changes are marked in the tracked-changes version of the manuscript (V1.docx).

| **Editor’s Comment** | **Author’s Response / Action Taken** |
| --- | --- |
| 1. Data Availability Requirement: All PLOS journals require that data underlying study findings be freely available, either (a) in a public repository, (b) within the manuscript, or (c) as supplementary information. | All relevant and de-identified programmatic data used for analysis (2017–2024) — including TB patient notification statistics, stakeholder engagement metrics, and Plan-Do-Check-Act (PDSA) cycle process documentation — have been provided as Supplementary File S1: “Akot TU TB Notification and PDSA Dataset.xlsx. This supplementary dataset enables full transparency and reproducibility, satisfying PLOS open-data requirements. |
| 2. Competing Interest Statement: We have amended your statement to comply with journal style; please confirm correctness. | Verified — the statement “*The authors declare that no competing interests exist*” is correct and remains unchanged |
| 3. Financial Disclosure Statement: Please clarify all funding sources and funder roles. Use exact publication wording. | The following statement is confirmed for publication: “The authors received no specific funding for this work. The funders had no role in study design, data collection and analysis, decision to publish, or preparation of the manuscript.” All authors agree. |
| 4. Citation of Reviewer-Recommended Works: Review and cite suggested works only if relevant. | All reviewer-suggested works were carefully reviewed. Relevant literature on PDSA methodology, digital TB notification frameworks, and public–private engagement (Refs 13–16) was incorporated; unrelated citations were not added. |

**Reviewer 1 Comments :**

| **Reviewer Comment** | **Author’s Response / Action Taken** |
| --- | --- |
| 1. Please consider reporting the annual TB case notification for Akot TU to show the TB incidence and proportion reported by private providers (e.g., 16 of 32 vs. 16 of 500). | Addressed. Annual TB notification data for Akot TU have now been added in Table 2 (2017–2025), reporting both public and private notifications with corresponding percentages. This allows readers to contextualize the intervention’s effect in relation to the total TB burden and notification trends in the area. |
| 2. Reporting both the number and proportion of TB case notifications from private providers will allow standardization and help distinguish true improvements from proportional changes. | Implemented. The Results section and Table 1 now include both absolute numbers and percentages of private TB notifications before and after intervention (16 of 211 cases [7.6%] vs. 63 of 211 [29.9%]), ensuring standardization and comparability across periods. |
| 3. Please report changes in TB notifications as changes in proportion of notifications from private providers as a fraction of all TB notifications. | The manuscript now expresses changes in private-sector notifications as both absolute and proportional increases, clearly showing the improvement attributable to the PDSA cycles. |
| 4. Line 46: Please provide an estimate of the size of the private provider network in the area to establish the denominator. | Added in Methods section: “Akot TU includes 191 private facilities — 52 IMA doctors, 65 NIMA practitioners, 14 laboratories, and 60 chemists — forming the denominator of stakeholders targeted during the intervention.” |
| 5. Line 46: Missing a word to go with ‘16’ (persons with TB? notifications? providers?). | Corrected for clarity: “16 TB notifications from private providers during January–July 2023.” |
| 6. Line 141: Need to capitalize the first word. | Corrected. Grammar and capitalization errors have been reviewed and resolved across the revised manuscript. |
| 7. Graph 2: Per the text, there were 16 + 63 = 79 TB notifications from private providers in 2023, but the figure stated 43. Please verify which is correct. | Verified with Ni-kshay data and corrected. The 2023 value is 79 private notifications, consistent across Table 2, Figure 1, and the text. All figures and statistical analyses were updated accordingly. |
| 8. If there were 79 notifications from 2023, the segmented regression needs recalculation. There might be a statistical difference now. | The Interrupted Time Series (ITS) analysis was re-run using updated 2023 data (79 cases). Results remain statistically significant for post-intervention trend (β₃ = +50.5, p = 0.032), confirming robustness. Updated regression coefficients are presented in Table 3 and the trend line revised in Figure 1. |
| 9. Figure 4: Suggest separating into two figures — (a) patient flow diagram and (b) text box with identified gaps. Merge entry boxes, mark barriers, use thinner arrows. | Addressed in full. Figure 4a (Patient Flow Pathway for TB Notification) now depicts a concise linear process with standardized arrow thickness and merged entry sources. Figure 4b lists identified barriers from PDSA cycles in bullet format for clarity and reader utility. |
| 10. Barriers can be signified by something blocking the flow of arrows; wording should be concise. | Implemented. Barriers are now visually shown beside the arrows with brief, one-line labels (e.g., “Missed referral,” “Delayed Ni-kshay entry”). Text simplified for readability. |
| 11. In your flow diagram, you have ‘TB treatment by PP’ as one of the outcomes. Were you able to assess whether they were on the correct treatment? | We thank the reviewer for this suggestion. Among 79 private notifications, 23 patients-initiated treatment under private providers. However, treatment appropriateness was not assessed, as it was beyond the study’s scope. This has been clearly acknowledged as a limitation in the Discussion section (Lines 367–371). |

**Reviewer 2 Comments**

| **Reviewer Comment** | **Author’s Response / Action Taken** |
| --- | --- |
| 1. Clarify the meaning of “private notifications accounted for 32% in 2017 and 90% by 2023.” Specify the denominator and contextualize these percentages. | We appreciate the reviewer’s insightful observation. The percentages refer to the proportion of private TB patient notifications out of total TB notifications under the National Tuberculosis Elimination Programme (NTEP). This has been clarified in the Introduction (Lines 81–86): “Private providers accounted for about 21% of total notified TB cases in 2017, increasing steadily to nearly 90% by 2023, according to Central TB Division data.” The revised text now explicitly mentions that these proportions represent private notifications as a share of total national TB notifications, not targets. |
| 2. The stated annual increase (4%) vs. 93% private notifications in 2023 appears inconsistent. Please reconcile this and clarify when significant improvement occurred (2024?). | Addressed in Results and Discussion sections. The non-significant annual increase (β₁ = +4.0) refers to Akot TU pre-intervention trend (2018–2022), whereas the national data (32% to 90%) describe the India-wide trend under NTEP (2017–2023). These represent different analytical contexts. The distinction has been clarified in the revised Discussion (Lines 356–367) and Introduction (Lines 81–90). The 2024 data for Akot TU now correctly show significant improvement post-intervention (private achievement 126%). |
| 3. Clarify the nature of the study—if described as a “quality improvement study,” the qualitative results (KIIs/FGDs) should be reported and linked with quantitative findings. | Fully incorporated. A dedicated “Challenges and Insights” subsection (Results, Lines 326–374) now presents key qualitative findings from Key Informant Interviews (KIIs) and field observations, explaining how they informed and reinforced the quantitative improvements. Triangulation between qualitative and quantitative results is explicitly described in the Discussion (Lines 364–385). |
| 4. Explain the term ‘Ni-kshay’ for global readers and ensure consistency (not ‘Nikshay’). | Corrected throughout the manuscript. The Introduction (Lines 86–94) now includes: “The term ‘Ni-kshay’ is derived from Sanskrit, where ‘Ni’ means free from and ‘Kshay’ means tuberculosis, symbolizing the goal of a TB-free India.” All instances have been standardized to “Ni-kshay.” |
| 5. Clarify the “universe” of facilities—how many health facilities existed, and what data sources were used. Address potential selection bias. | Addressed comprehensively in Methods – Study Area and Population (Lines 115–142). We now describe how the complete mapping of 191 private facilities was achieved using multiple sources: Ni-kshay provider database, District Health Office records, Clinical Establishment Registry, IMA/NIMA membership lists, and field verification by the PPM Coordinator. This triangulated mapping minimized selection bias. Coverage completeness is explicitly discussed. |
| 6. Explain why regularly notifying stakeholders were excluded from the study. What was the rationale for distinguishing under-reporters? | Added justification in Methods (Lines 142–155). Regular notifiers were excluded intentionally to focus the quality improvement intervention on underperforming providers. Regular notifiers were defined as those reporting ≥1 TB case in ≥3 of the past 4 quarters. Including them could have diluted measurable intervention effects. This rationale aligns with quality improvement principles and ensures focus on low-performing segments. |
| 7. Add a flow diagram summarizing the number and type of stakeholders, inclusions/exclusions, and follow-up details. | Implemented. Figure 2 (Patient Flow Pathway and Barriers in TB Notification) and Figure 3 (Stakeholder Inclusion and Process Flow Diagram) have been added. These illustrate the stepwise inclusion of stakeholders: total facilities (191) → Ni-kshay registered (118) → newly identified (73) → sensitized (90) → followed up monthly (25). |
| 8. Figures (Graph 1, Graph 2) lack proper titles and captions per journal style. | Corrected. All graphs and figures now have descriptive, self-contained captions per PLOS formatting (e.g., *Figure 1. Segmented regression showing annual trend of private TB patient notifications in Akot TU, 2018–2024*). |
| 9. Throughout the manuscript, “TB notification” should be changed to “TB patient notification” for precision, since notification may also refer to TB deaths or preventive therapy. | Revised throughout. The term “TB patient notification” is now used consistently across the title, abstract, main text, and figures to avoid ambiguity. |

**Reviewer 3 comments**

| **Reviewer Comment** | **Detailed Author Response / Action Taken** |
| --- | --- |
| 1. Insufficient post-intervention periods: The authors use only one post-intervention time point, which is inadequate for ITS regression analysis. Multiple post-intervention periods are essential for proper trend assessment. A difference-in-difference approach with explicit time factors may be more appropriate for this data structure. | We sincerely thank the reviewer for this valuable comment. The initial submission indeed included limited post-intervention data (November–December 2023), which constrained the temporal robustness of the Interrupted Time Series (ITS) model. In the revised version, we have expanded the analysis period to include data through December 2024, sourced from verified Ni-kshay notifications. This extension allowed us to incorporate fourteen post-intervention monthly data points, providing sufficient temporal granularity to detect sustained changes in private-sector TB patient notifications. The model now captures both the immediate level change (β₂) following the intervention and the post-intervention slope change (β₃) across a full calendar year. The text in the Methods (Lines 182–195) and Results (Lines 285–307) has been updated accordingly. Additionally, we have included a note in the Limitations (Lines 400–405) acknowledging that despite this extension, longer follow-up may further enhance the precision of trend estimates. This methodological strengthening improves internal validity and ensures compliance with standard ITS design requirements. |
| 2. Inappropriate distribution: The authors appear to use Gaussian regression for count data. Poisson or negative binomial regression with population offset would be more suitable for this data type. | We fully acknowledge this point and have implemented the recommended correction. The revised model employs segmented Poisson regression with a population offset, which is statistically appropriate for count-based event data such as TB notifications. The population size of the Akot Tuberculosis Unit (approximately 280,000) was included as an offset variable to standardize notification rates. Model estimation was performed in R (version 4.3.2) using robust standard errors to adjust for potential overdispersion. This specification replaces the earlier Gaussian model, thereby enhancing accuracy in estimating rate ratios and temporal effects. The equation is now explicitly presented in the Methods section (Lines 192–195) as: *log(Yₜ) = β₀ + β₁(Timeₜ) + β₂(Interventionₜ) + β₃(PostInterventionTrendₜ) + log(Populationₜ)*. These revisions ensure that the statistical analysis aligns with best practices in epidemiological modelling of count outcomes. Furthermore, the model’s improved fit (Adjusted R² = 0.78) and lower residual variance have been reflected in the Results (Table 3) and Discussion (Lines 358–365). |
| 3. Model specification unclear: The regression parameters lack clarity. Including the actual model equation rather than describing it as "segmented regression" would improve transparency and reproducibility. | This important suggestion has been fully implemented. The complete model structure and equation are now presented in the Methods (Lines 192–195) and accompanied by a paragraph explaining the interpretation of each term—baseline trend (β₁), immediate intervention effect (β₂), and post-intervention slope (β₃). We have also added a justification for using segmented Poisson regression to account for temporal autocorrelation and potential level shifts. The parameter definitions and coefficients are clearly displayed in Table 3, where each β term’s magnitude and 95% confidence interval are reported. |
| 4. Temporal reporting inconsistencies: The description contains multiple conflicting time periods that create confusion about the study timeline. | We thank the reviewer for drawing attention to this issue. All time periods have now been systematically standardized across the manuscript to eliminate inconsistencies. The corrected chronology is as follows:   - Baseline period: January–July 2023 - Intervention phase (PDSA cycles): August–October 2023 - Immediate post-intervention evaluation: November–December 2023 - Extended post-intervention monitoring: January–December 2024   These standardized timelines are now consistently reflected in the Methods (Lines 150–165), Results (Table 1, Table 2, Figure 1), and Discussion (Lines 345–350). The figure captions and data tables have been revised accordingly to ensure that time references correspond precisely to the intervention and evaluation phases. |
| 5. These methodological concerns significantly impact the validity of the conclsions drawn | We deeply appreciate this constructive critique and have undertaken a comprehensive revision of the analytical framework to address each methodological concern systematically. Specifically, the post-intervention period was extended, the statistical model was changed to a segmented Poisson regression with offset, the model equation was explicitly defined for reproducibility, and all time-related inconsistencies were corrected. Furthermore, the Discussion (Lines 368–385) now explicitly contextualizes these improvements, emphasizing that the strengthened analysis reinforces the robustness and credibility of the study’s conclusions. The Limitations section (Lines 400–406) transparently acknowledges that future research with longer post-intervention follow-up and multi-site replication could provide additional validation. Overall, these substantial analytical refinements now ensure that the conclusions are both methodologically soud and globally relevant for quality improvement in TB surveillance. |
