# Supplementary material for: Improving private tuberculosis patient notifications in Akot Tuberculosis Unit of Vidarbha region of India: A Plan-Do-Study-Act (PDSA) based approach for enhanced public health reporting
Source: PLOS Glob Public Health. 2026 May 22;6(5):e0006480. doi: 10.1371/journal.pgph.0006480 (PMC13196973; doi:10.1371/journal.pgph.0006480)
Supplement: Attachment — Submitted filename: reply to reviwer comment.docx [file pgph.0006480.s003.docx]

**Reply to reviewer comment**

**Manuscript ID: PGPH-D-25-01093**

Title: Improving Private Tuberculosis (TB) Notifications in Akot Tuberculosis Unit of Vidarbha Region of India: A Plan-Do-Study-Act (PDSA) Based Approach for Enhanced Public Health Reporting

Journal: PLOS Global Public Health

Corresponding Author: Dr Umesh G. Kawalkar

Email: umeshkawalkar01@gmail.com

We thank the Editor and all reviewers for their detailed critique and constructive feedback. All comments were reviewed meticulously. Substantial revisions were made to ensure internal consistency, data accuracy, improved readability, and alignment with PLOS Global Public Health standards. Changes are marked in the tracked-changes version of the manuscript.

| **Reviewer** | **Comment** | **Author Response** |
| --- | --- | --- |
| **Reviewer 1** | **Line 237: What was the total pool of low-reporting private providers? This would provide the denominator for the 90 that attended the sensitization.** | We thank the reviewer for this important clarification. The follow-up component of the intervention was not limited to participants of the sensitization workshops, nor was workshop attendance intended to represent the denominator of low-reporting providers. All private healthcare facilities in Akot TU were comprehensively mapped (n = 191), and regularly notifying providers were excluded. The Public–Private Mix (PPM) coordinator conducted targeted monthly follow-ups with non-notifying providers irrespective of workshop attendance. The Methods section has been revised to clearly distinguish between system-wide sensitization activities and targeted follow-up of under-reporting providers, thereby resolving denominator ambiguity. |
| **Reviewer 1** | **Inaccuracy in using 211 as denominator for both pre- and post-intervention periods. Period-specific denominators should be used.** | We agree with the reviewer. The annual total was inappropriate for sub-period comparisons. Table 1 has been revised to use period-specific denominators, recalculating proportions as 16/90 (17.8%) for Jan–Jul 2023 and 63/121 (52.1%) for Nov–Dec 2023. The Results text has been updated accordingly. As anticipated by the reviewer, the post-intervention proportion increased when correct denominators were applied. |
| **Reviewer 2** | **The manuscript has improved substantially. Clarification of statistical analysis and interpretation would strengthen the paper.** | We thank the reviewer for the positive assessment. We have revised the Methods, Results, and Discussion sections to clarify the statistical analyses, particularly the interrupted time-series (ITS) methodology, coefficient interpretation, and associated limitations. |
| **Reviewer 2** | **No further major concerns.** | We sincerely appreciate the reviewer’s constructive feedback and support, which have helped improve the rigor and clarity of the manuscript. |
| **Reviewer 3** | **ITS implementation has fundamental problems: monthly vs annual mismatch.** | We thank the reviewer for this methodological point. Although month-wise data were available for selected periods, complete and consistent monthly notification data across the full study period were not available. We therefore conducted an annual ITS analysis using segmented regression. This choice and its limitation are now clearly stated in the Methods section. |
| **Reviewer 3** | **Population offset concerns; implausible coefficients; unclear units.** | We agree. The ITS model has been re-specified as a segmented linear regression using annual counts, without population offsets. Coefficients are now interpreted as changes in annual counts and trends rather than rates. All interpretations have been revised accordingly. |
| **Reviewer 3** | **Model specification unclear; post-trend coding not explained.** | We thank the reviewer for highlighting this. The post-intervention trend has now been explicitly parameterized as time elapsed since intervention onset ((T − T_i)), consistent with recommended ITS methodology. This specification is clearly described in the Methods section. |
| **Reviewer 3** | **COVID-19 pandemic not incorporated.** | We agree. The COVID-19–affected years (2020–2021) are now explicitly acknowledged as important contextual influences in the Methods and Discussion sections. Findings are interpreted cautiously in light of these disruptions. |
| **Reviewer 3** | **Seasonality should be examined if monthly data exist.** | We acknowledge this limitation. Formal assessment of seasonality was not feasible due to incomplete month-wise data. This limitation is now explicitly stated in the Discussion section. |
| **Reviewer 3** | **Overall interpretation implies causality rather than association.** | We agree and have moderated causal language throughout the manuscript. All ITS and chi-square findings are now consistently interpreted as associations rather than causal effects. |
| **Reviewer 3** | **Table 1 conflates association with causation.** | We thank the reviewer and agree. All causal language has been removed from the Results and Discussion. Table 1 now explicitly reports associations only. |
| **Reviewer 3** | **Chi-square results not reproducible; incorrect denominator (211).** | We agree. Table 1 has been corrected using period-specific denominators (90 and 121). The 2×2 table was reconstructed and chi-square statistics recalculated, resulting in a reproducible and methodologically correct analysis. |
| **Reviewer 3** | **Table presentation requires clarification.** | Table 1 has been revised to clearly define row and column categories, simplify statistical reporting to a single Pearson’s chi-square test, and include a footnote stating that the test assesses association and not causation. |
| **Reviewer 3** | **Table 2 trends need better interpretation; pre-2019 rise, COVID decline, 2022 recovery ignored.** | We agree and have revised the Results section to explicitly describe improvements during 2017–2019, the COVID-19-related decline in 2020–2021, and recovery in 2022 prior to intervention implementation. |
| **Reviewer 3** | **Attributing changes solely to intervention ignores contextual factors.** | We fully agree. The Discussion now explicitly states that findings should be interpreted in the context of pre-existing trends, COVID-19 disruptions, and concurrent TB programme modifications rather than attributed solely to the intervention. |
| **Reviewer 3** | **Qualitative methods lack detail: who collected data, when, who interviewed, what observations were done.** | We have expanded the Qualitative Methods section to specify the data collection period (March–October 2023), data collectors (authors and PPM coordinator), purposive participant selection, interviewee categories, and structured field observations conducted during implementation. |
| **Reviewer 3** | **Qualitative results report challenges but not observational findings.** | We agree and have added an explicit subsection reporting observational findings, demonstrating how field observations corroborated and contextualized interview-based themes. |
| **Reviewer 3** | **Manuscript organization mixes Methods, Results, and Discussion.** | We thank the reviewer for this observation. The manuscript has been comprehensively reorganized to ensure clear separation of Methods (procedures), Results (quantitative and qualitative findings), and Discussion (interpretation and contextualization). |
