# Supplementary material for: Improving private tuberculosis patient notifications in Akot Tuberculosis Unit of Vidarbha region of India: A Plan-Do-Study-Act (PDSA) based approach for enhanced public health reporting
Source: PLOS Glob Public Health. 2026 May 22;6(5):e0006480. doi: 10.1371/journal.pgph.0006480 (PMC13196973; doi:10.1371/journal.pgph.0006480)
Supplement: Attachment — Submitted filename: Response to Reviewers.docx [file pgph.0006480.s004.docx]

**Reply to reviewer comment**

**Manuscript ID: PGPH-D-25-01093**

Title: Improving Private Tuberculosis (TB) Notifications in Akot Tuberculosis Unit of Vidarbha Region of India: A Plan-Do-Study-Act (PDSA) Based Approach for Enhanced Public Health Reporting

Journal: PLOS Global Public Health

Corresponding Author: Dr Umesh G. Kawalkar

Email: umeshkawalkar01@gmail.com

We thank the Editor and all reviewers for their detailed critique and constructive feedback. All comments were reviewed meticulously. Substantial revisions were made to ensure internal consistency, data accuracy, improved readability, and alignment with PLOS Global Public Health standards. Changes are marked in the tracked-changes version of the manuscript.

**Response**

| **Comment** | **Response** | **Changes made in manuscript** |
| --- | --- | --- |
| **Editor** | | |
| Reviewers have raised concerns about analytic method. Please address or describe limitations clearly if data constraints prevent further analysis. | We thank the editor for the valuable suggestion. Due to the absence of complete month-wise notification data prior to the intervention, a longer monthly baseline or formal counterfactual modelling was not feasible. To address this, we incorporated long-term annual trend analysis (2017–2024) using interrupted time-series segmented regression and explicitly described analytic constraints as study limitations. Findings are now interpreted as associations rather than causal effects. | Methods – Data Analysis section revised; Limitations paragraph expanded; Discussion updated to clarify interpretation of associations. |
| **Reviewer 1** |  |  |
| Why were the first 7 months of 2023 used as baseline? Would 2022 stabilize baseline? | January–July 2023 was selected because it immediately preceded the intervention (August 2023) and therefore best represents the operational context immediately prior to intervention initiation. To provide broader context, long-term notification trends from 2017–2024 have now been analysed and presented using interrupted time-series methods. | Baseline justification added in Methods; annual trend analysis (2017–2024) included in Results (Tables and ITS analysis). |
| Without the intervention, would 2023 counts have been projected differently? | Because month-wise pre-intervention data were unavailable, formal counterfactual projections could not be reliably estimated. This methodological limitation is now explicitly described, and results are interpreted as associations rather than causal effects. | Limitations section expanded and interpretation clarified in Discussion. |
| Any possibility of including private notifications from 2024? | Yes. Annual notification data for 2024 have now been included descriptively to illustrate continuation of trends, while causal interpretation remains limited to the defined analysis period. | Table showing year-wise notifications updated to include 2024 data. |
| **Reviewer 2** |  |  |
| All comments addressed. | We sincerely thank the reviewer for the constructive feedback and confirmation that the revisions adequately addressed earlier comments. | Minor language refinements performed throughout the manuscript. |
