# Supplementary material for: Improving private tuberculosis patient notifications in Akot Tuberculosis Unit of Vidarbha region of India: A Plan-Do-Study-Act (PDSA) based approach for enhanced public health reporting
Source: PLOS Glob Public Health. 2026 May 22;6(5):e0006480. doi: 10.1371/journal.pgph.0006480 (PMC13196973; doi:10.1371/journal.pgph.0006480)
Supplement: Attachment — Submitted filename: response to comment.docx [file pgph.0006480.s005.docx]

**Dear Dr. Graeme Hoddinott,**

Thank you for the opportunity to revise and resubmit our manuscript titled **“Improving Private Tuberculosis Patient Notifications in Akot Tuberculosis Unit of Vidarbha region of India: A Plan–Do–Study–Act (PDSA) Based Approach for Enhanced Public Health Reporting.”**

We sincerely appreciate the thoughtful comments provided by you and the reviewer. The methodological concerns raised regarding the analytical approach and interpretation of results were extremely valuable in improving the clarity and transparency of our study.

In revising the manuscript, we carefully reviewed the issues highlighted, particularly those related to the **interrupted time-series analysis, the intervention timeline, and the interpretation of notification trends**. Where appropriate, we have clarified methodological details, expanded the description of the intervention context, and revised the discussion to ensure that the findings are interpreted cautiously within the framework of a real-world quality-improvement initiative.

We respectfully note that the study was conducted as a **programmatic quality-improvement intervention within routine National Tuberculosis Elimination Programme activities**, which limited the ability to retrospectively redesign the evaluation framework. Nevertheless, we have made substantial revisions to improve transparency, acknowledge limitations, and clarify the analytical approach used.

We hope that these revisions adequately address the concerns raised and improve the overall rigor and interpretability of the manuscript. A detailed point-by-point response to all comments is provided below, and all revisions have been clearly indicated in the tracked-changes version of the manuscript.

We thank you again for your careful consideration of our work.

Warm regards,

**Dr. Umesh Kawalkar**
Corresponding Author
Department of Community Medicine
Government Medical College, Washim
Maharashtra, India

**Responses to Editorial Comment**

| **Comment** | **Response** | **Changes Made in Manuscript** |
| --- | --- | --- |
| **Editorial Comment** | | |
| **Comment 1: Concerns were raised during previous review rounds regarding the methodological approach used in the study, particularly the interrupted time-series (ITS) analysis.** | We thank the editor for highlighting these important methodological considerations. In response, we carefully revisited the analytical approach and clarified the purpose and interpretation of the interrupted time-series analysis in the manuscript. We emphasize that the ITS analysis was intended primarily to illustrate **long-term contextual trends in private TB notifications**, rather than to establish causal inference regarding the intervention. | The Methods section has been revised to clarify the analytical framework used for ITS analysis. Additional explanation has been added in the Discussion section to ensure appropriate interpretation of the results. |
| **Comment 2: While the topic of the manuscript was considered relevant, some reviewers expressed uncertainty regarding aspects of the analytical methods and their interpretation.** | We appreciate this observation and have therefore expanded the methodological description to improve transparency and clarity. Additional details have been provided regarding the **intervention timeline, data sources, baseline definition, and interpretation of statistical analyses**. These revisions were intended to make the analytical approach clearer for readers. | The Methods section has been expanded to provide clearer descriptions of the intervention timeline, Ni-kshay data sources, and analytical approach. |
| **Comment 3: The analysis was suggested to be reconsidered or clarified in light of the reviewer comments before the manuscript could be considered further.** | We carefully reviewed the reviewer comments and addressed each concern in detail. We respectfully clarify that the study was conducted as a **programmatic quality-improvement initiative embedded within routine tuberculosis control activities; therefore, the evaluation reflects operational program implementation rather than a controlled experimental design**. Accordingly, the analysis was designed to evaluate programmatic changes in notification patterns rather than causal effects. The manuscript has been revised to clarify this implementation context and to acknowledge analytical limitations. | The Methods, Discussion, and Limitations sections have been revised to clearly state the programmatic nature of the study and avoid causal over-interpretation. |

**Responses to Reviewer #4**

| **Comment** | **Response** | **Changes Made in Manuscript** |
| --- | --- | --- |
| **Comment 1:** The authors note that “Stakeholders who had already been regularly notifying TB cases were excluded to focus efforts on those with the highest potential for change.” Are the TB notification data reported limited to the stakeholders included in the intervention, or do they reflect data from all private practitioners? If the latter, it should be clarified that the intervention targeted certain stakeholders but data from all stakeholders were included in the analysis. | We thank the reviewer for this important clarification. The intervention activities (workshops, digital outreach, and follow-up visits) were targeted primarily toward under-reporting private providers identified during the mapping exercise. However, the outcome data on TB notifications were extracted from the **Ni-kshay platform**, which captures notifications from **all private healthcare providers within the Akot Tuberculosis Unit jurisdiction**, not only those who participated directly in the intervention. Thus, while the intervention targeted selected stakeholders, the analysis reflects system-level notification trends across the entire private provider network within the TU. | The **Methods section** has been revised to clearly state that notification data obtained from the Ni-kshay platform include **all private providers within the TU jurisdiction**, while the intervention targeted under-reporting providers. |
| **Comment 2:** The intervention included linking private providers to Nikshay who were not previously linked. The pre- and post-intervention periods therefore include different sets of providers who could report TB notifications. The post-intervention period includes a higher number of linked providers, which on its own could lead to an increase in notifications. | We appreciate the reviewer’s observation. As part of the intervention, a comprehensive mapping exercise identified several private healthcare providers who were not previously registered on the Ni-kshay platform. These providers were subsequently sensitized and linked to the system during the intervention period. We acknowledge that expansion of the registered provider pool may partly contribute to the increased notifications observed in the post-intervention period. The manuscript has therefore been revised to clarify that the observed improvements likely reflect a **combined influence of provider linkage, sensitization activities, digital engagement, and supportive programmatic follow-up**, rather than the intervention alone. | Additional clarification has been added in the **Methods section** describing provider mapping and linkage to the Ni-kshay platform. The **Discussion and Limitations sections** have also been revised to acknowledge that expanded provider linkage may partly influence notification trends. |
| **Comment 3:** It appears that 90 TB notifications were reported during the 7-month pre-intervention period (Jan–Jul 2023), whereas 121 cases were reported during the 2-month post-intervention period (Nov–Dec 2023). This suggests possible temporal trends in case notifications. The pre- and post-intervention periods should ideally cover the same calendar months to allow better comparison (e.g., Nov 2022–Jul 2023 vs Nov 2023–Jul 2024). | We thank the reviewer for this helpful suggestion. The baseline period (January–July 2023) was intentionally defined as the **immediate operational context preceding the intervention**, while the intervention was implemented between **August and October 2023** through iterative PDSA cycles. The post-intervention period (November–December 2023) therefore represents the **first complete operational phase following implementation**, allowing evaluation of the immediate programmatic effect of the intervention. Because the study was conducted as a **real-world quality-improvement initiative embedded within routine programme implementation**, extending the baseline or post-intervention periods to other calendar windows could introduce unrelated contextual influences such as programme changes or seasonal variation. To address broader temporal trends, we supplemented the pre-post comparison with **longitudinal trend analysis using interrupted time-series methods**. | The **Methods section** has been revised to clarify the rationale for defining the baseline and post-intervention periods based on the intervention timeline. |
| **Comment 4:** The ITS analysis time periods do not appear appropriate. The private TB notification counts are very low from 2018–2022 and then increase sharply from 2023 onward. It may therefore be more appropriate to focus on shorter time intervals (e.g., monthly or bi-monthly data from 2022–2024) when assessing trends. | We thank the reviewer for this valuable methodological suggestion. While interrupted time-series analyses ideally use shorter intervals such as monthly data, historical notification data for earlier years (2017–2021) were available only in **annual aggregated format within district programme records**, which limited the feasibility of performing a consistent monthly ITS analysis across the entire study period. The annual ITS analysis was therefore used to illustrate **long-term contextual trends in private TB notifications**, including the disruption observed during the COVID-19 pandemic and the subsequent recovery phase. We acknowledge that annual aggregation limits analytical granularity; therefore, we have clarified in the revised manuscript that the ITS results should be interpreted as **supportive contextual trend analysis rather than causal inference**. | Additional clarification has been added in the Methods section describing the use of annual notification data, and the Discussion (Limitations subsection) has been expanded to acknowledge the constraints related to incomplete month-wise historical data and interpretation of ITS results. |
